# Disease control and disease activity in hereditary angioedema: two sides of the same coin?

**DOI:** 10.3389/fimmu.2025.1631448

**Published:** 2025-07-22

**Authors:** Behzad Heibati, Jack Borle, Bruce Ritchie, Adil Adatia

**Affiliations:** ^1^ Division of Pulmonary Medicine, Department of Medicine, University of Alberta, Edmonton, AB, Canada; ^2^ Faculty of Science, University of Alberta, Edmonton, AB, Canada; ^3^ Division of Hematology, Department of Medicine, University of Alberta, Edmonton, AB, Canada

**Keywords:** hereditary angioedema, C1 inhibitor, patient reported outcomes, immunodeficiency, angioedema

## Abstract

**Background:**

Hereditary angioedema (HAE) is a genetic disorder characterized by episodic subcutaneous and submucosal swelling. Patient-reported outcome measures (PROMs) are recommended for routine clinical assessment by international guidelines and are used as secondary outcome measures in pivotal clinical trials for novel investigational drugs. The Angioedema Control Test (AECT) and Hereditary Angioedema Activity Score (HAE-AS) are validated tools designed to aid in HAE patient assessment, but the extent to which they measure different disease outcomes is unclear. The aim of this study was to examine how these measures correlate and inform clinical practice.

**Methods:**

We conducted a retrospective study of patients with HAE types 1 and 2 at the Edmonton Angioedema Center of Reference and Excellence. AECT and HAE-AS scores were obtained from patient-completed questionnaires during routine visits. Multiple linear regression was used to examine the association between HAE-AS and AECT scores with age, sex, and long-term prophylaxis (LTP) status as predictors. Receiver operating characteristic (ROC) analysis was used to determine the optimal HAE-AS score cutoff that predicts poor disease control as determined by the AECT.

**Results:**

There were 25 participants included with a mean age of 39.4 years (SD = 13.7), 72% of whom were female. Most had HAE Type 1 (76%) and 52% were receiving LTP. SC C1-inhibitor therapy was the most common LTP (36%). Most had well managed disease with a median AECT score of 11.88 (range: 5-16) and HAE-AS of 5.84 (range: 0-13). A statistically significant but weak negative correlation was found between AECT and HAE-AS (β=−0.67, p=0.002). ROC analysis showed that an HAE-AS score >5 had a sensitivity of 100% and specificity of 61% for poor disease control.

**Conclusions:**

The AECT and HAE-AS instruments are weakly correlated, indicating that they provide related but distinct information to the practicing clinician. Using both AECT and HAE-AS in clinical practice can thus provide a more comprehensive patient evaluation.

## 1 Introduction

Hereditary angioedema due to C1 inhibitor deficiency (HAE) is a rare genetic condition caused by mutations in the gene *SERPING1* (1, 2). Patients present with episodic submucosal and skin swelling affecting the limbs, oropharynx, face, bowels, and genitals. Swelling results from aberrant bradykinin generation owing to loss of the inhibitory function of the *SERPING1* gene product C1 inhibitor (C1-inh) on plasma kallikrein (3, 4). There are two subtypes of HAE. Type 1 is caused by the reduction in the amount of C1-inh produced and type 2 is caused by the production of nonfunctional C1-inh (5, 6).

Clinical guidelines for the management of HAE emphasize the use of validated patient reported outcome measures (PROMs) to ensure careful assessment of each patient and to guide management decisions (2, 7, 8). Registration trials of HAE treatments also use PROMs as secondary outcomes to ensure these metrics improve with reduced angioedema attack frequency and severity (9–11). Several instruments have been developed including the AECT (12–14), HAE-AS (15), angioedema activity score (16), and the angioedema quality of life score (17). Some are specific to HAE and others apply broadly to all recurrent angioedema syndromes.

Some PROMs aim to measure disease activity whereas others aim to measure disease control (18). Disease activity refers to the ongoing intensity and frequency of symptoms during a defined period. It is a measure of how manifest the disease is, typically reflected in the number of angioedema attacks, their severity, and their impact on normal function (15, 19). Disease control refers to the extent to which the disease is prevented from manifesting or is stabilized, often because of treatment. It assesses how well therapeutic interventions are working in reducing or preventing angioedema attacks and in restoring normal function. Disease control is not simply the absence of symptoms but reflects an overall assessment of how well the disease is being managed.

The AECT and the HAE-AS are two validated instruments that measure disease control and activity, respectively (20, 21). The AECT is also used as a secondary outcome measure in clinical trials for investigational products for HAE (22, 23). It is unclear how these instruments correlate with each other and whether they provide similar or complementary information to the practicing clinician. Herein, we report a cross-sectional study conducted to understand the relationship between these two metrics.

## 2 Methods

### 2.1 Study design

We conducted a retrospective study of patients with HAE types 1 and 2 seen at the Edmonton Angioedema Center for Reference and Excellence (ACARE) between April 1, 2022, and April 1, 2023 (24). In this time period, both AECT and HAE-AS were administered at patient visits as part of clinical care. Patients were included if they had a confirmed diagnosis of HAE type 1 or 2, were aged ≥18 years, and had completed both questionnaires.

### 2.2 Study procedures

Patients completed the AECT and HAE-AS questionnaires during their clinic visits. Data were collected from patient records, including completed questionnaire scores and relevant clinical information.

### 2.3 HAE-AS score

The HAE-AS is an instrument specifically developed to measure the activity of angioedema in patients with HAE. It is comprised of 12 items, of which 7 pertain to attacks occurring over the previous 6 months, and 5 pertain to emergency visits, psychological status, days of school/work missed, impairment in work/activities due to pain, and general health. Each item is scored from 0 to 3, where higher scores indicate higher disease activity over a recall period of six months (21). Most items use a 6-month recall period, except for the questions pertaining to general health and impairment in daily activities, which use a 1-month period (20). The raw score is transformed into a linear measure, resulting in a sum score ranging from 0 to 30. A raw HAE-AS <12 indicates mild or low activity, while a score of 13 or higher indicates severe disease activity (20).

### 2.4 AECT score

The AECT is a 4-item PROM developed to retrospectively assess disease control over time in patients with recurrent angioedema from any cause. Each AECT item offers five answer options, scored from 0 to 4 points (16, 21). The four AECT items assess subjective attack frequency, impact, treatment effectiveness, and unpredictability. There are two versions of AECT: one with a 4-week recall period and another with a 3-month recall period. In this study, we used the version with a 3-month recall period. The total AECT score was computed by summing the scores of all four items, yielding a range from 0 (poor disease control) to 16 (excellent disease control), with higher scores indicating better angioedema control. Scores ≥ 10 were considered to indicate well-controlled disease.

### 2.5 Statistical analysis

Descriptive statistics were calculated to summarize baseline characteristics of the study population. Continuous variables, such as age, were presented as means with standard deviations (SD) for normally distributed data. Categorical variables, including sex, disease type, and long-term prophylaxis use, were reported as frequencies and percentages. To examine the association between the AECT score and HAE-AS, a scatter plot with a fitted regression line was created, and the correlation was assessed using both Pearson’s and Spearman’s correlation coefficients, as the normality of the AECT score was borderline. To better understand the contribution of each individual item, we also examined the association between each AECT question and linearized HAE-AS. Associations were considered weak if <0.4, moderate if 0.4 ≤ < 0.6, and strong if ≥ 0.6.

The concordance between AECT score and HAE-AS was defined as AECT showing poor control (score <10) and HAE-AS showing high activity (score ≥12), or AECT showing good control (score ≥10) and HAE-AS showing low activity (score <12). The proportion of concordant observations was calculated, with results expressed as a percentage of the total. Receiver operating characteristic (ROC) analysis was also performed and Youden’s J (25) was calculated to determine the optimal HAE-AS cutoff that indicates poor disease control (AECT ≥ 10). To assess differences in AECT scores between participants with and without LTP, we employed a Welch two-sample t-test. A multiple linear regression model was used to assess the association between linearized/raw HAE-AS as the dependent variable and AECT score, age, sex, and LTP status as predictors. A generalized additive model (GAM) with spline was used to model the non-linear association between AECT and log-transformed HAE-AS, stratified by LTP status. Statistical significance was defined as p<0.05. All statistical analyses were performed using R (version 4.3.2).

## 3 Results

The characteristics of the study population are summarized in **Table 1**. The mean age was 39.4 years (SD = 13.7), and most were female (72%) and had HAE Type 1 (76%). LTP was utilized by 52% of the participants. SC C1-inhibitor therapy was the most common LTP, administered to 36% of participants. IV C1-inhibitor therapy and berotralstat were each used by 8% of participants. Of the 25 study participants, most had well-controlled disease according to their AECT scores. The mean (SD) AECT score was 11.88 (3.44), but 7 (28%) had an AECT score below 10.

**Table 1:** Demographics of study population (n = 25).

Characteristic	
**Age (years), mean (SD)**	39.44 (13.71)
Sex, n (%)	
Male	7 (28.0)
Female	18 (72.0)
Disease, n (%)	
Type 1	19 (76)
Type 2	6 (24)
Long-term prophylaxis, n (%)	
No	12 (48.0)
Yes	13 (52.0)
**Berotralstat, n (%)**	2 (8)
**IV C1 inhibitor, n (%)**	2 (8)
**SC C1 inhibitor, n (%)**	9 (36)
**AECT score, mean (SD)**	11.88 (3.44)
**HAE-AS, mean (SD)**	5.84 (3.53)

IV, intravenous; SC, subcutaneous; AECT, Angioedema control test; HAE-AS, Hereditary Angioedema activity score; SD, standard deviation.

The results of the HAE-AS showed low disease activity among the participants. The mean raw HAE-AS (SD) was 5.84 (3.53) and the mean score for the linearized HAE-AS (0–30 scale) was 9.54 (SD = 4.43). Only, one participant (4%) had a raw score >12 and they also had a low AECT score of 9. Overall, 76% (19/25) of participants were classified as having concordant AECT and HAE-AS scores; the 24% with discordant scores all had AECT scores <10 but HAE-AS scores also <12. The ROC analysis showed that the HAE-AS score > 5 was optimal cutoff to predict poor disease control (AECT scores <10) with a sensitivity of 100% and a specificity of 61.1%.

**Figure 1:**
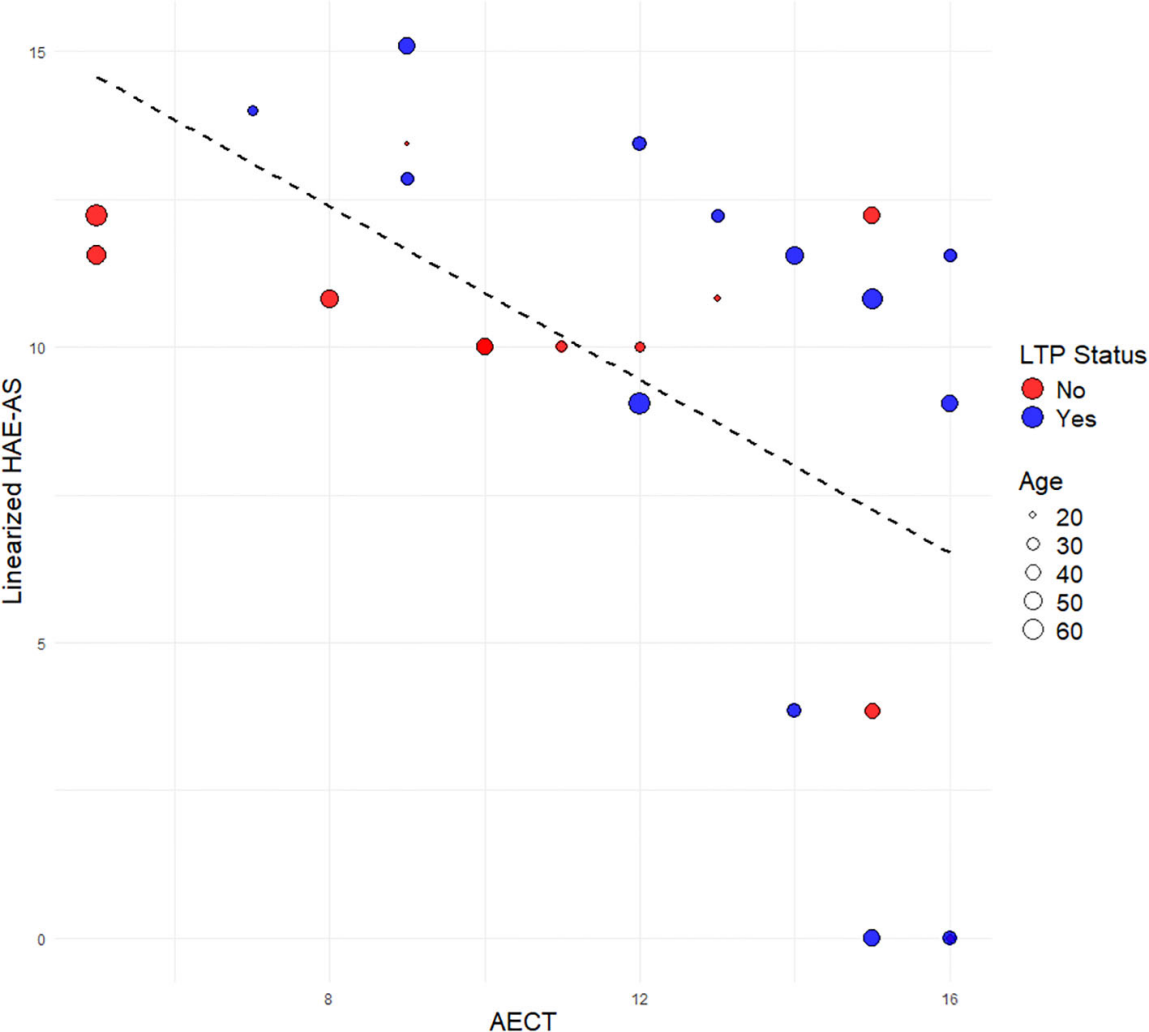
Linear association between AECT and linearized HAE-AS by age and LTP status.

Pearson correlation analysis showed a statistically significant negative correlation between AECT and the raw HAE-AS (r=−0.57, p=0.002). A similar moderately strong correlation was observed when using the linearized HAE-AS scale (r = −0.57, p = 0.003) (**Figure 1**). This indicates that as AECT score increases, HAE-AS tends to decrease. The association was consistent across individuals with HAE Type 1 and Type 2. Age did not appear to significantly influence this association, as there was no observable clustering of age within specific ranges of AECT Scores or HAE-AS.

The strength of correlation between the linearized HAE-AS and the mean scores of individual AECT questions varied (**Supplementary Figure S3**). Question 1 on the perception of attack frequency (“In the last 3 months, how often have you had angioedema?”) had the greatest negative correlation (r = –0.59). Questions 2 (“In the last 3 months, how much has your quality of life been affected by angioedema?” and 3 (“In the last 3 months, how much has the unpredictability of your angioedema bothered you?”) had similar coefficients of correlation (r = –0.48 and r = –0.49, respectively) indicating moderate association. However, there was a weak correlation (r = –0.26) with question 4 (“In the last 3 months, how well has your angioedema been controlled by your therapy?”).

Univariate analysis showed that the mean AECT score was higher in the LTP group (12.92) compared to the non-LTP group (10.75), but the difference was not statistically significant (p=0.12). Similarly, no significant difference in HAE-AS was observed between males and females (p=0.11), although males had a numerically higher mean HAE-AS (7.57) compared to females (5.17). There was a negative association between AECT and HAE-AS, with both male and female patients and across ages (**Supplementary Figure S1**).

In the multiple linear regression model using raw HAE-AS, AECT score (β=−0.67, p=0.002) showed a statistically significant negative association with HAE-AS, indicating that for each unit increase in AECT score, HAE-AS decreased by approximately 0.67 units (data not shown). LTP status (β=2.97, p=0.03) was also significantly associated with HAE-AS, with participants receiving LTP having an average HAE-AS score 2.98 units higher than those not on LTP, after adjusting for the other variables. Age (β=−0.04, p=0.27) and sex (β=−1.59, p=0.30) were not significant predictors in this model.

Using the linearized HAE-AS scale, the AECT score (β = −0.75, p = 0.01) remained a statistically significant negative predictor of disease activity (**Table 2**) in the multiple linear regression model. This suggests that for each unit increase in AECT score, the linearized HAE-AS score decreased by approximately 0.75 units, indicating better angioedema control is associated with lower disease activity. LTP status (β = 2.37, p = 0.19) showed a positive but non-significant association with the linearized HAE-AS score, suggesting that patients on LTP had, on average, 2.37 units higher HAE-AS scores than those not receiving LTP, though this did not reach statistical significance. Age (β = −0.01, p = 0.74) and sex (β = −1.75, p = 0.40) were not significant predictors in this model.

**Table 2:** Multivariable linear regression analysis of predictors of linearized HAE-AS (0-30).

Predictor	Estimate	SE	p-value
AECT	-0.75	0.26	0.01*
Age	-0.01	0.05	0.74
Sex (Female)	-1.75	2.06	0. 40
LTP (Yes)	2.37	1.76	0.19

* indicates statistical significance at the alpha level of 0.05.

**Supplementary Figure S2** shows the spline-modeled association between AECT scores and log-transformed HAE-AS, stratified by LTP status. Across both groups, higher AECT scores are associated with lower predicted disease activity. Notably, the steepest decline in HAE-AS occurs beyond AECT > 12, suggesting a possible threshold effect with a greater association between high AECT and improved disease activity in that range.

## 4 Discussion

This cross-sectional study investigated the association between two validated HAE PROMs: the AECT, which assesses disease control, and the HAE-AS, which measures disease activity. The main findings are a) the AECT and HAE-AS are moderately correlated indicating that they provide related but distinct information about patient clinical status, b) the AECT is most sensitive to HAE-AS when AECT >12, suggesting that even low disease activity significantly worsens patient-perceived disease control, and c) a threshold of HAE-AS > 5 appears to imply poor disease control.

We found a moderate association between the AECT and HAE-AS scores in HAE patients. This finding reinforces that disease control and disease activity represent complementary but distinct constructs and the utility of incorporating multidimensional assessment tools in HAE management to guide individualized treatment strategies and improve patient outcomes. The non-linear association showed a potential threshold effect, with the most pronounced reduction in HAE-AS occurring with AECT scores >12. This finding suggests that, compared to the asymptomatic state, small increases in disease activity can significantly impair patient perception of disease control.

These data are consistent with the existing literature showing that even a single attack can impair quality of life (26, 27). For example, the HELP study, which evaluated the efficacy of lanadelumab and used the Angioedema Quality of Life instrument (AE-QoL) to measure disease-related quality of life, found that 44.4% of patients in the lanadelumab 300 mg q2w group were attack-free and exhibited a mean AE-QoL improvement of 31.2 points from baseline over 26 weeks (9). However, patients with ≥1 attack showed a mean improvement of 15.1 points, which is less than half the improvement compared to attack-free patients, and attack-free status was associated with 2.1-fold greater likelihood of achieving the minimal significant difference threshold compared to non-attack-free patients (28).

Question 4 of the AECT, which assesses perceived effectiveness of treatment, was only weakly associated with the HAE-AS. This may be because patients consider the improvement in disease activity achieved by their current treatment regimen rather than the current disease activity when answering the question. As several new HAE medicines for both on demand treatment and LTP are in development or expected to be marketed imminently (29), even those scoring well on this Question may therefore still benefit from switching to a new agent.

Nearly a quarter of patients had poor disease control using the AECT but low disease activity according to the recommended HAE-AS threshold of 12 points. This discordance was observed despite that the mean linearized HAE-AS score in our study (9.54 ± 4.43) is comparable to the value reported in previous prospective multicenter cohort study of 290 adult patients (10.66 ± 3.92) (20), indicating consistency in symptom burden across the two cohorts using the same interval transformation method. We posit that factors captured in the AECT such as fear of having an attack and reduced self-efficacy realized from the patient’s perception of incomplete treatment response explain why low disease activity can be seen with poor AECT scores. Qualitative investigational approaches examining these patients’ disease and treatment experiences would be useful to evaluate this hypothesis. We additionally explored alternate thresholds using ROC analysis and found that a HAE-AS score >5 was the optimal cutoff to detect poor disease control in our sample with excellent sensitivity, though specificity was modest.

Individuals on LTP in our study consistently demonstrated higher predicted HAE-AS scores compared to those not on LTP, indicating a persistently elevated disease burden despite similar AECT scores. This difference may reflect confounding by indication, as patients receiving LTP likely represent those with more severe or refractory disease. It also highlights that LTP was frequently not sufficient to achieve the guideline recommended treatment target of complete control (1), at least in this cohort in which plasma-derived C1 inhibitor was the predominant LTP agent used.

Children were not included in the present study since these instruments are not validated in the pediatric population (26). The lack of such tools, which are critical for both interventional studies of novel therapeutics and for routine clinical assessment, represent a major unmet need. Approximately one-half of HAE patients first become symptomatic by age 10 years (30). Pointedly, childhood onset of HAE attacks is associated with adverse outcomes in multiple domains including psychosocial development, mental health, educational attainment, and vocational success (31). Without validated instruments, these outcomes cannot be adequately assessed and monitored.

There are limitations to this study. As a single-center study in a well-resourced country, there was a high prevalence of modern LTP use and consequently the HAE-AS scores were relatively low. There were, however, no subjects treated with lanadelumab or garadacimab included, which limits the generalizability of our findings to populations in which these are the predominant agents. Future larger, multicenter studies may allow for a broader comparison between HAE-AS and AECT scores by recruiting patients with higher disease activity and allow for assessment of treatment responsiveness of each instrument. The sex distribution was skewed towards females, which is a near universal finding in HAE studies (9, 11, 20, 32–34) and likely reflects the increased disease severity (and thus higher rate of diagnosis and study participation) amongst females; in the present study this may have resulted in higher AECT and HAE-AS scores but is unlikely to have affected the correlation between the two instruments. Older adults were not represented in the cohort and thus the findings may not apply to the geriatric population. The study is also limited by small sample size though comparable in size to other studies of PROs in HAE (14). We were also unable to account for the differing recall periods used by the AECT and HAE-AS.

## 5 Conclusion

In this study, a moderate negative correlation was observed between disease control and disease activity scores, as measured by the AECT and HAE-AS suggesting that they capture complementary dimensions of disease burden. Future longitudinal studies are needed to better understand how AECT and HAE-AS scores evolve over time and in response to treatment modifications, and to validate their combined utility in guiding clinical decision-making.

## Data Availability

The raw data supporting the conclusions of this article will be made available by the authors, without undue reservation.

## Supplementary material

Supplementary Figure 1 Association between AECT and linearized HAE-AS by sex and LTP status.

Supplementary Figure 2 Spline-modeled association between AECT and logDAS by LTP status.

Supplementary Figure 3 Association between individual AECT question and linearized HAE-AS by age and LTP status (Q1: frequency of swelling in the past 3 months, Q2: quality of life in the past 3 months, Q3: burden of unpredictability in the past 3 months, Q4: perceived control of angioedema with current therapy in the past 3 months).
