# Supplementary figures and images for: Disease control and disease activity in hereditary angioedema: two sides of the same coin?
Source: Front Immunol. 2025 Jul 22;16:1631448. doi: 10.3389/fimmu.2025.1631448 (PMC12321560; doi:10.3389/fimmu.2025.1631448)

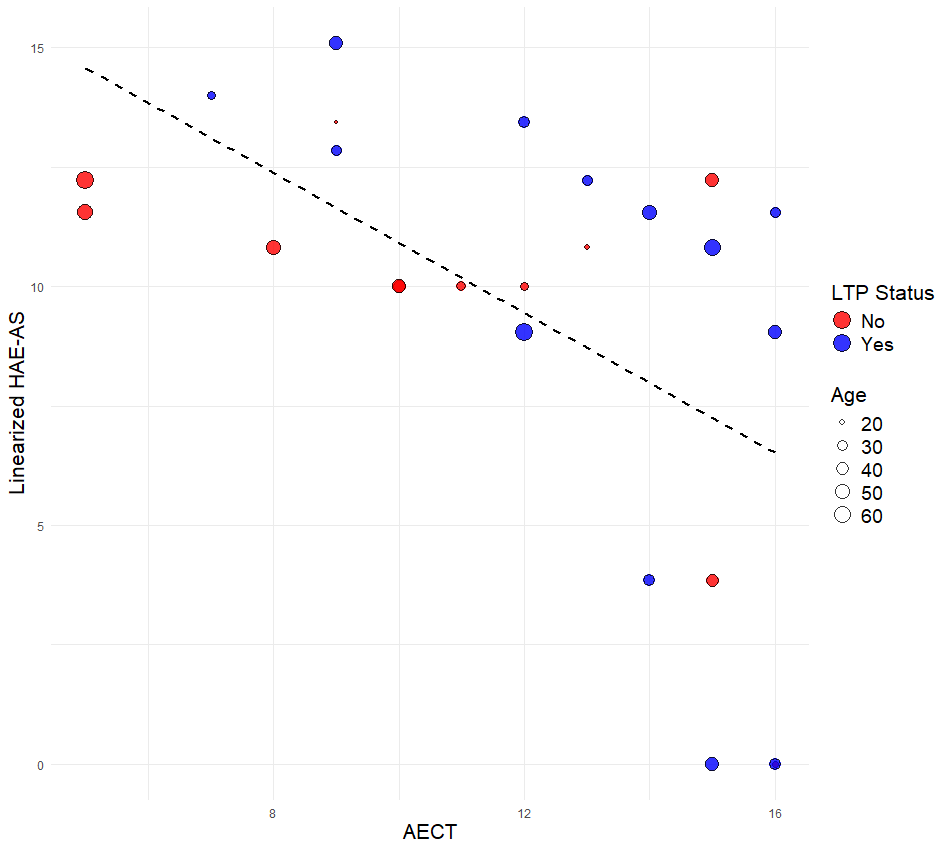

Supplement: Supplementary Figure 1 — Association between AECT and linearized HAE-AS by sex and LTP status. [file Image1.tiff]

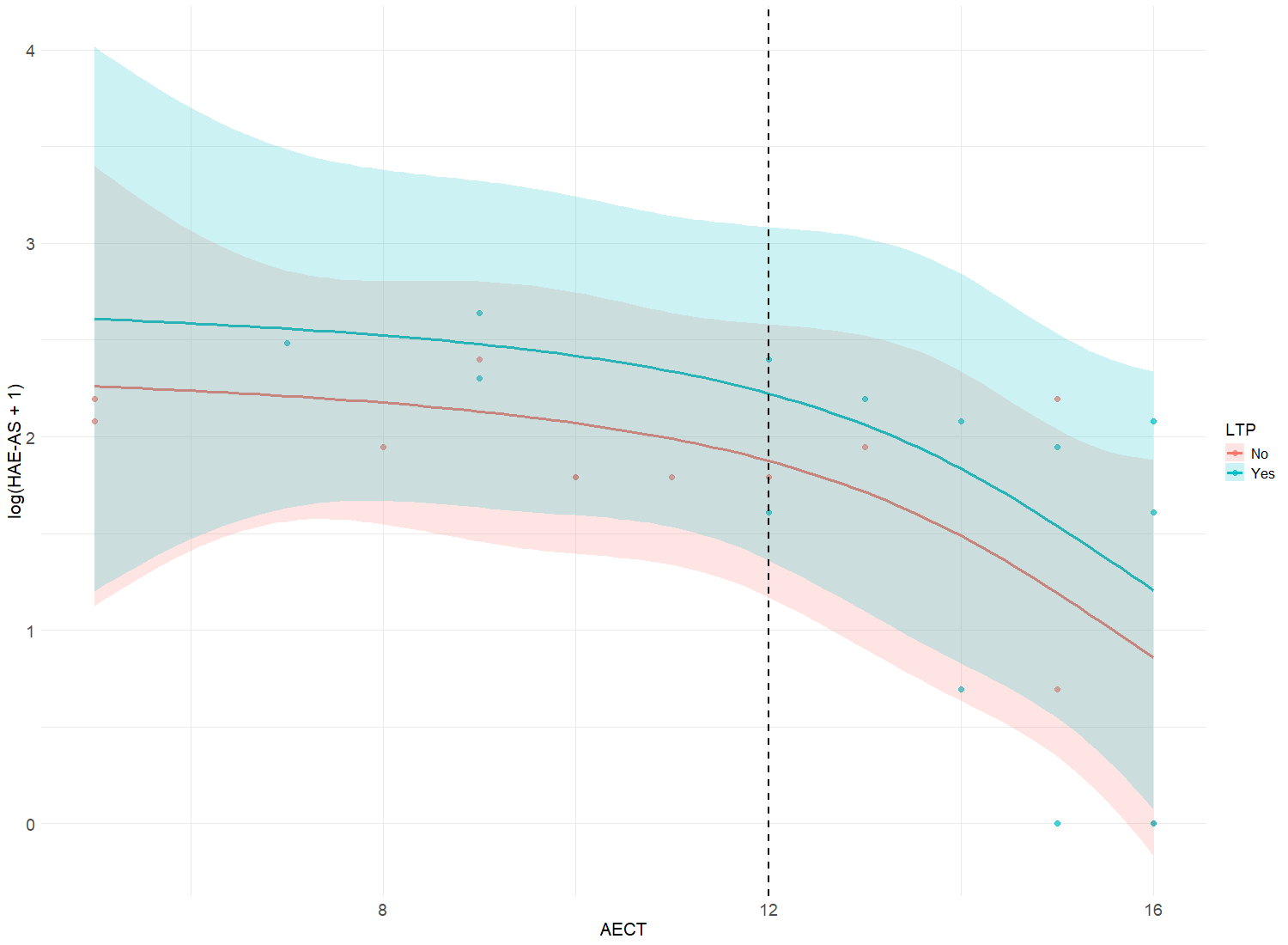

Supplement: Supplementary Figure 2 — Spline-modeled association between AECT and logDAS by LTP status. [file Image2.tiff]

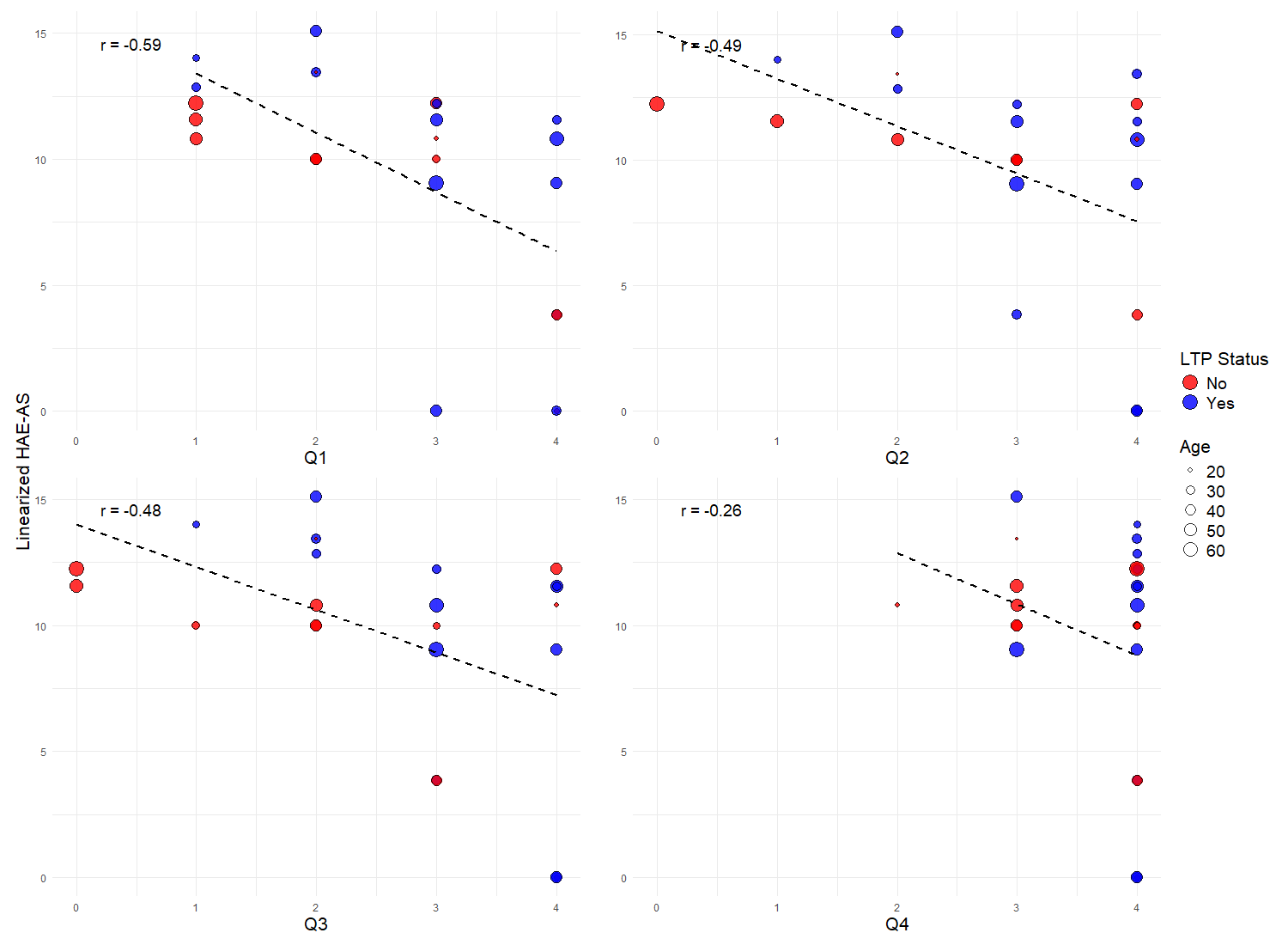

Supplement: Supplementary Figure 3 — Association between individual AECT question and linearized HAE-AS by age and LTP status (Q1: frequency of swelling in the past 3 months, Q2: quality of life in the past 3 months, Q3: burden of unpredictability in the past 3 months, Q4: perceived control of angioedema with current therapy in the past 3 months). [file Image3.tiff]
